# Pulmonary Hypertension: Let’s Take Stock!

**DOI:** 10.3390/life15071137

**Published:** 2025-07-18

**Authors:** Michele Cacia, Egidio Imbalzano, Vincenzo Antonio Ciconte, Marco Vatrano

**Affiliations:** 1 Cardiology Unit, Azienda Ospedaliero Universitaria “Renato Dulbecco”, P.O. “Pugliese”, 88100 Catanzaro, Italy; cacia.michele@gmail.com (M.C.); enzocico2003@yahoo.it (V.A.C.); 2 Department of Clinical and Experimental Medicine, University of Messina, 98125 Messina, Italy; egidio.imbalzano@unime.it

**Keywords:** pulmonary hypertension, rare disease, treatments

## Abstract

Pulmonary hypertension (PH) encompasses a group of conditions characterized by elevated pulmonary arterial pressure, with pulmonary arterial hypertension (PAH) representing a distinct and severe subset. This review provides a comprehensive overview of the current classification system, highlighting the five clinical groups of PH and the specific hemodynamic criteria defining PAH. We discuss the complex pathophysiological mechanisms underlying PAH, including vascular remodeling, endothelial dysfunction, and genetic predisposition. Advances in diagnostic approaches are explored. Current treatment strategies targeting key molecular pathways such as endothelin, nitric oxide, and prostacyclin are reviewed alongside novel and investigational therapies. Prognostic indicators and risk stratification tools are evaluated to guide clinical management. Finally, we underscore the critical role of expert centers in accurate diagnosis, multidisciplinary care, and enrollment in clinical trials, which collectively improve patient outcomes in this challenging disease spectrum.

## 1. Introduction

**Figure 1:**
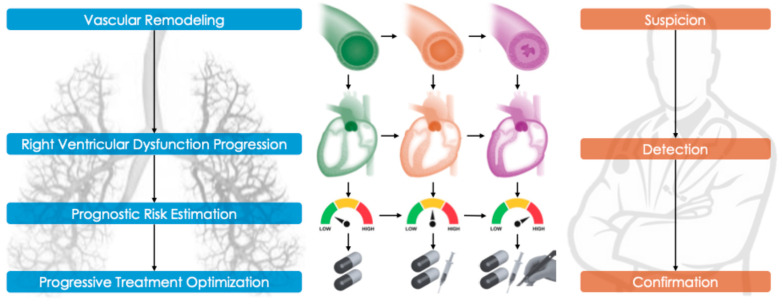
The figure illustrates the progression of pulmonary hypertension from a pathophysiological perspective and the conceptual basis of the diagnostic approach, with the aim of stratifying annual mortality risk and, consequently, the type of pharmacological treatment (oral, parenteral, and transplant-based).

Pulmonary hypertension (PH) is a rare condition in its idiopathic form but occurs more frequently as a secondary manifestation of other cardiovascular or respiratory diseases. It is characterized by non-specific symptoms and a progressive increase in pulmonary vascular resistance (PVR), which can lead to dysfunction—often irreversible—of the right heart chambers. Pulmonary arterial hypertension (PAH), a specific subset of PH, involves only the arterial component of the pulmonary vasculature. A correct and early diagnosis is necessary to assess the risk of annual mortality, to decide the type of treatment to start, and to determine its subsequent optimization during the short-term follow-up (Figure 1). In this comprehensive review, we aim to summarize the latest evidence on the disease, from its definition to current and emerging treatment options.

## 2. Epidemiology

PH affects approximately 1% of the global population, with a prevalence of up to 10% among individuals over 65 years of age. The female-to-male ratio is approximately 4.8:1 [1], and PH is present in at least 50% of patients with heart failure (HF) [2]. According to earlier epidemiological data, the French registry [3] estimated the prevalence of PAH at 15 cases per million inhabitants, with an incidence of 2.4 cases per million per year. The Scottish registry [4], by contrast, reported a prevalence ranging from 26 to 52 cases per million and an incidence between 7.1 and 7.6 cases per million annually. More recent registries, including SPAHR (2019) [5], the UK National Health Service Audit (2019) [6], the Polish Registry of Pulmonary Hypertension (BNP-PL, 2018) [7], the Latvian Registry (2016) [8], and the Comparative, Prospective Registry of Newly Initiated Therapies for Pulmonary Hypertension (COMPERA, 2014) [9], as well as others [10,11], indicate that PAH prevalence and incidence estimates range from 12.4 to 268 cases per million (ppm) and from 1.5 to 32 ppm per year, respectively. For chronic thromboembolic pulmonary hypertension (CTEPH), the estimated prevalence and incidence in adults range from 14.5 to 144 ppm and from 0.9 to 39 ppm, respectively [12]. Pediatric epidemiological data on PAH are limited [13,14], with the reported incidence ranging from 2.4 to 16.7 ppm and prevalence from 3.7 to 397 ppm.

## 3. Pathophysiology and Disease Mechanisms

PH is now recognized as a multifactorial vascular disease involving a complex interplay of cellular and molecular abnormalities. Traditional paradigms of PAH pathobiology—such as endothelial dysfunction, imbalance between vasoconstrictors and vasodilators, and unchecked vascular cell proliferation—have been reinforced by recent evidence [15]. In PAH, pulmonary artery endothelial cells exhibit the reduced production of vasodilators, including nitric oxide (NO) and prostacyclin, alongside the increased synthesis of vasoconstrictive and mitogenic mediators such as endothelin-1. This endothelial dysfunction promotes chronic vasoconstriction and stimulates the proliferation of adjacent vascular smooth muscle cells and fibroblasts via a cascade of growth factors and cytokines. Notably, PAH lesions display the overexpression of growth factors (e.g., platelet-derived growth factor [PDGF], fibroblast growth factor-2) and pro-inflammatory cytokines, which drive the remodeling of the vascular wall and extracellular matrix. A major breakthrough in our understanding of PAH was the discovery of genetic mutations predisposing individuals to the disease, particularly heterozygous loss-of-function mutations in the bone morphogenetic protein receptor type 2 (BMPR2) gene. BMPR2 signaling normally acts as a key inhibitor of vascular cell proliferation; over 80% of heritable PAH cases and approximately 20% of idiopathic PAH cases carry BMPR2 mutations. Disruption of BMPR2 signaling—either through genetic mutation or acquired downregulation—removes a critical restraint on cell growth and promotes a pro-inflammatory state within the pulmonary vasculature. Conversely, upregulation of the transforming growth factor-beta (TGF-β) signaling pathways, such as those involving activin A and ALK1, has been implicated as a complementary mechanism driving vascular remodeling in PAH. Chronic inflammation and immune dysregulation are now acknowledged as central contributors to the pathogenesis of various forms of PH. In PAH associated with connective tissue diseases (e.g., scleroderma), pulmonary arteries often exhibit intense inflammatory infiltrates. However, even idiopathic and heritable forms of PAH demonstrate signs of immune activation. Elevated circulating levels of cytokines—including interleukin-6 (IL-6), interleukin-1β, and others—are commonly found in PAH patients and correlate with disease severity. These cytokines can directly promote the proliferation of pulmonary artery smooth muscle and endothelial cells, and their expression is often increased in the context of BMPR2 mutations. Moreover, autoimmune features—such as circulating autoantibodies and perivascular inflammatory cell aggregates—have been identified even in idiopathic PAH, suggesting the existence of a spectrum of immune-driven disease phenotypes. Recent research has proposed the existence of distinct immune phenotypes in PAH, which may eventually inform personalized immunomodulatory treatment strategies. Beyond inflammation, several additional mechanisms have been implicated in the vascular remodeling seen in PH, including metabolic and mitochondrial dysfunction in the right ventricle and pulmonary vasculature, dysregulated apoptosis and autophagy, and epigenetic alterations such as DNA methylation and changes in microRNA expression. Together, these discoveries underscore that PH—particularly PAH—is not merely a disease of elevated pulmonary pressure, but a complex vascular disorder driven by genetic, inflammatory, metabolic, and proliferative mechanisms.

## 4. Classification and Hemodynamic Diagnosis of Pulmonary Hypertension

**Figure 2:**
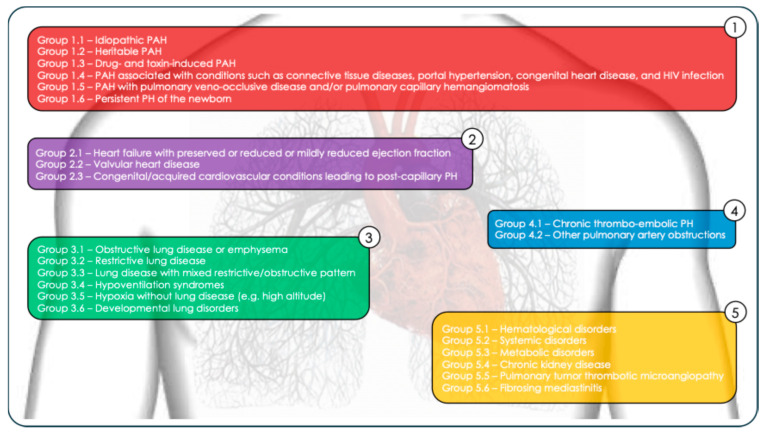
The WHO classification of pulmonary hypertension.

The World Health Organization (WHO) classifies PH into five clinical groups based on etiology (Figure 2). The most common causes of PH are cardiac diseases (Group 2) and respiratory diseases (Group 3). PH due to left heart disease (PH-LHD) results from elevated left atrial pressure, typically secondary to conditions such as heart failure with preserved or reduced ejection fraction, or valvular heart disease. Patients with PH-LHD may present with either isolated post-capillary PH—defined by a pulmonary artery wedge pressure (PAWP) > 15 mmHg and PVR ≤ 2 Wood Units (WU)—or combined pre- and post-capillary PH, characterized by PAWP > 15 mmHg and PVR > 2 WU [16]. PH secondary to chronic lung disease and/or hypoxia (PH-CLD) is linked to a reduction in the pulmonary vascular bed, decreased vascular distensibility, and impaired recruitment of the pulmonary vessels. This form is commonly associated with chronic obstructive pulmonary disease (COPD), interstitial lung disease, and sleep apnea syndrome [17]. Pulmonary arterial hypertension (PAH, Group 1), although the rarest form, is the most severe in terms of its prognosis and represents the only subgroup currently treatable with pulmonary vasodilator therapies (excluding post-thromboembolic forms in Group 4). Group 1 PAH is further divided into several subtypes:

- Idiopathic PAH (Group 1.1);
- Heritable PAH (Group 1.2);
- Drug- and toxin-induced PAH (Group 1.3);
- PAH associated with conditions such as connective tissue diseases, portal hypertension, congenital heart disease, and HIV infection (Group 1.4);
- PAH with pulmonary veno-occlusive disease and/or pulmonary capillary hemangiomatosis (Group 1.5);
- Persistent PH of the newborn (Group 1.6).

Chronic thromboembolic PH (CTEPH, Group 4) arises from the persistent obstruction of the pulmonary arteries by organized thromboembolic material (Group 4.1), most often following acute pulmonary embolism or other causes of pulmonary artery obstruction (Group 4.2). The resulting vascular remodeling contributes to the chronic increase in pulmonary pressure [18]. Group 5 includes cases of PH with unclear or multifactorial mechanisms. This group encompasses the following:

- Hematological disorders (Group 5.1: chronic hemolytic anemia, myeloproliferative diseases);
- Systemic disorders (Group 5.2: sarcoidosis, pulmonary Langerhans cell histiocytosis, neurofibromatosis type 1);
- Metabolic disorders (Group 5.3: glycogen storage diseases, Gaucher disease)
- Chronic kidney disease (Group 5.4);
- Pulmonary tumor thrombotic microangiopathy (Group 5.5);
- Fibrosing mediastinitis (Group 5.6).

The definitive diagnosis of PH is made via right heart catheterization (RHC). This invasive procedure is essential not only to confirm the diagnosis but also to differentiate PAH (Group 1) from other forms. PH is defined hemodynamically as a mean pulmonary arterial pressure (mPAP) > 20 mmHg at rest, measured during RHC.

The most recent guidelines from the European Society of Cardiology (ESC) [19] have lowered the diagnostic threshold for mPAP from 25 mmHg to 20 mmHg, acknowledging that mPAP values between 21 and 24 mmHg are associated with an increased risk of adverse outcomes. This early-stage hemodynamic profile has been termed “early PH.” Importantly, classification also relies on PAWP and PVR to define the hemodynamic subtype of PH:

○ Pre-capillary PH (e.g., PAH, CTEPH): mPAP > 20 mmHg, PAWP ≤ 15 mmHg, PVR > 2 WU;
○ Isolated post-capillary PH (due to left heart disease): mPAP > 20 mmHg, PAWP > 15 mmHg, PVR ≤ 2 WU;
○ Combined pre- and post-capillary PH: mPAP > 20 mmHg, PAWP > 15 mmHg, PVR > 2 WU (as seen in heart failure patients with associated pulmonary vascular disease).

## 5. Diagnostic Workup of Suspected Pulmonary Hypertension

**Figure 3:**
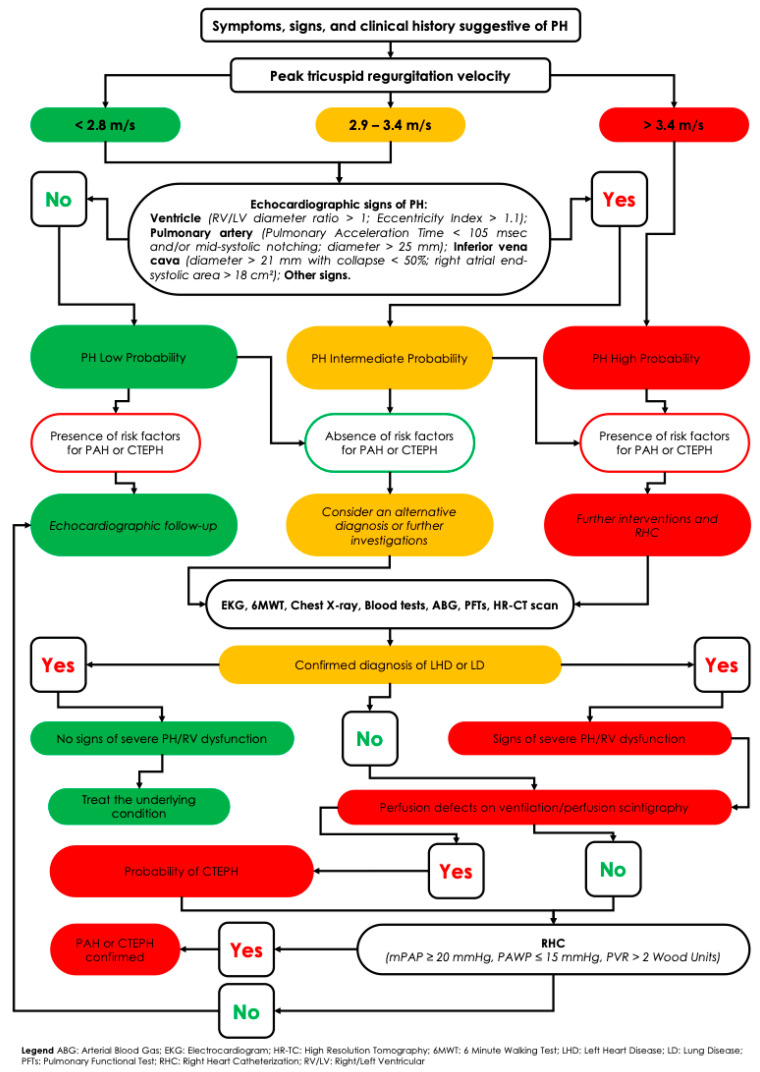
The diagnostic workup of pulmonary hypertension.

When PH is suspected, a comprehensive first-line assessment should include the following:

- Clinical evaluation, with a thorough history and physical examination aimed at identifying common symptoms such as exertional dyspnea, fatigue, weakness, angina, fluid retention (e.g., ankle edema), and syncope.
- Transthoracic echocardiography, the cornerstone of initial screening, provides an estimate of the probability of PH. Although it offers derived measurements (e.g., right ventricular systolic pressure), it also supplies valuable hemodynamic, morphological, and functional data—such as right atrial and ventricular dilation, pericardial effusion, deviation of the interventricular septum (indicative of right-sided pressure overload), systolic and diastolic ventricular function, and the presence of valvular disease.
- Additional investigations to determine the underlying cause may include the following:
  ○ Chest radiography;
  ○ Pulmonary function tests (spirometry, body plethysmography, and diffusing capacity for carbon monoxide [DLCO]);
  ○ Arterial blood gas analysis;
  ○ Sleep studies or overnight oximetry;
  ○ High-resolution computed tomography (HRTC);
  ○ Laboratory tests.

- Ventilation/perfusion (V/Q) scintigraphy, essential in all patients without confirmed left heart or primary lung disease, to rule out chronic thromboembolic pulmonary hypertension (CTEPH). Advanced imaging techniques such as single-photon emission computed tomography (SPECT V/Q) and dual-energy computed tomography (DECT) enhance the detection of perfusion defects, even in distal pulmonary vessels [20]. SPECT V/Q offers 3D perfusion imaging with improved sensitivity for detecting chronic thromboembolic obstructions while preserving high specificity. A normal V/Q scan effectively rules out CTEPH, whereas multiple segmental perfusion defects with preserved ventilation are diagnostic. If the V/Q scan is suggestive, confirmatory pulmonary angiography—either conventional (invasive) or CT-based—is required to localize and map thromboembolic lesions [21].
- DECT pulmonary angiography, for example, produces color-coded perfusion maps by tracking iodine distribution, combining high-resolution anatomical visualization with functional assessment in a single scan. Studies have shown good concordance between DECT perfusion imaging and V/Q scintigraphy, particularly in identifying small peripheral defects often missed by conventional CT [22].
- Cardiopulmonary magnetic resonance imaging (MRI) has also advanced significantly in PH diagnostics. Dynamic contrast-enhanced perfusion MRI enables the real-time visualization of lung perfusion without ionizing radiation. Recent studies report that optimized perfusion MRI achieves a sensitivity of ~95–97% for CTEPH diagnosis, comparable to V/Q scintigraphy. Non-contrast MRI techniques, such as phase-resolved functional lung imaging (PREFUL MRI), have demonstrated >90% agreement with V/Q SPECT in identifying regional perfusion abnormalities. Although MRI is not yet a first-line tool—mainly due to limited availability and cost—it offers a promising radiation-free alternative for patients ineligible for nuclear imaging and may become more widely used as technology evolves [22].
- Right heart catheterization (RHC) remains the definitive test to confirm the presence and type of PH—pre-capillary, post-capillary, or combined—particularly after non-invasive tests have excluded alternative diagnoses. RHC is indispensable for obtaining accurate pulmonary hemodynamic parameters, including mPAP, PAWP, cardiac output, and PVR. It also plays a key role in risk stratification and is mandatory before initiating targeted PAH therapy.
- Additional Investigations for Etiological and Prognostic Assessment:
  ○ Genetic testing and counseling are recommended in cases of idiopathic, heritable, or drug/toxin-induced PAH, as outlined in the 2022 ESC/ERS guidelines.
  ○ Mutations in the BMPR2 gene are found in approximately 75% of heritable PAH cases and over 25% of idiopathic PAH [23].
  ○ If BMPR2 testing is negative, further evaluation for mutations in ALK1 and ENG (associated with hereditary hemorrhagic telangiectasia, or Rendu–Osler–Weber syndrome), as well as other genes such as SMAD9, CAV1, and KCNK3, is warranted.
  ○ Genetic evaluation is also advised in patients with clinical or radiological suspicion of pulmonary veno-occlusive disease (PVOD), particularly to detect autosomal recessive EIF2AK4 mutations recently linked to heritable forms of PVOD [24].
- Biomarkers, such as brain natriuretic peptide (BNP) and N-terminal pro-brain natriuretic peptide (NT-proBNP), provide important prognostic information and help assess right ventricular strain (Figure 3).

## 6. Therapeutic Strategy and Follow-Up in PAH

**Figure 4:**
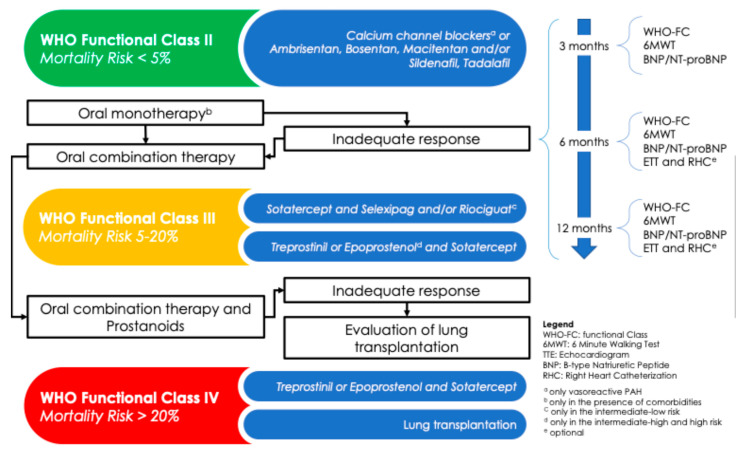
Therapeutic strategy and follow-up of pulmonary arterial hypertension.

The relentless progression of PAH, despite the availability of targeted therapies, necessitates periodic clinical and prognostic reassessment, as recommended by current guidelines. This ongoing evaluation is essential to optimize the therapeutic approach and achieve effective and sustained disease control. During follow-up, it is crucial to identify patients who are at high risk of mortality as early as possible, in order to escalate treatment using more potent, complex, and effective pharmacological agents. When indicated and not contraindicated, referral for lung transplantation should also be considered. PAH types included in WHO Groups 2 to 5 are secondary to other conditions, and their management is primarily based on treating the underlying disease mechanism. Therefore, the use of PAH-specific therapies is reserved exclusively for Group 1 PAH and is guided by a comprehensive, multiparameter risk assessment. A tailored treatment with calcium channel blockers (CCBs) is indicated only in patients who demonstrate a positive acute vasoreactivity test, which is defined as a reduction in mPAP of ≥10 mmHg to a value ≤ 40 mmHg, without a concomitant decrease in cardiac output. In these responders, high-dose CCBs—such as nifedipine, amlodipine, or diltiazem—are strongly recommended. However, their use does not preclude the addition of other targeted therapies based on the patient’s clinical status. Currently, several drug classes are approved for PAH treatment (Figure 4), and they may be used as monotherapy or in combination:

- Endothelin receptor antagonists (ERAs): ambrisentan, bosentan, macitentan;
- Phosphodiesterase type 5 inhibitors (PDE5is): sildenafil, tadalafil;
- Soluble guanylate cyclase stimulators (sGCSs): riociguat;
- Prostacyclin analogs (PAs): beraprost, epoprostenol, treprostinil;
- Prostacyclin receptor agonists (PRAs): selexipag.

A major objective of the recent revision of the hemodynamic definition of PH is to facilitate earlier diagnosis and the more timely initiation of therapy. Indeed, studies have estimated a mean delay of approximately 2.8 years between symptom onset and confirmed diagnosis [25]. Survival rates remain variable, ranging from 68% to 93% at one year, and from 39% to 77% at three years [1], underscoring the importance of early detection and risk-adapted treatment strategies.

## 7. Risk Stratification

At the time of diagnosis and before starting therapy, individualized risk stratification with the 1-year estimation of mortality rate must be performed to initiate the correct therapeutic approach. Furthermore, PH patients should be followed on a regular basis and current guidelines strongly suggest re-stratifying the risk during the follow-up to tailor the therapy. Two different multiparametric models to stratify the risk are adopted.

- At the time of diagnosis, a three-strata model is recommended, in which the variables to take in account are as follows: sign of right HF, progression of symptoms, presence of syncope, the WHO functional class, 6 min walking distance (6MWD), biomarkers (BNP or NT-proBNP), echocardiographic parameters (right atrium area, TAPSE/PAP ratio, pericardial effusion), magnetic resonance indexes (right ventricular ejection fraction, stroke volume index, right ventricular end-systolic volume index), and hemodynamic values after RHC (right atrial pressure, cardiac index, stroke volume index, mixed venous oxygen saturation). Patients can be classified at low risk (<5%), intermediate risk (5–20%), or high risk (>20%).
- At the follow-up, a simplified four-strata model including 6 min walking distance, BNP or NT-proBNP, and WHO functional class should be adopted, although additional variables like right heart imaging and hemodynamics parameters can be included. This model can divide patients into four categories: low risk, intermediate–low risk, intermediate–high risk, and high risk.

Personal and not negligible factors such as sex, age, comorbidities, and disease type should be considered at any time of evaluation.

### 7.1. Group 1

The latest European Guidelines recommend high-dose CCB therapy for patients with idiopathic, heritable, or drug-induced pulmonary arterial hypertension (PAH) who demonstrate a positive response to acute vasoreactivity testing (Class I, level of evidence C). In non-vasoreactive patients without cardiopulmonary comorbidities, mortality risk should be assessed. For patients with low to intermediate risk, initial combination therapy with a PDE5i and an ERA is recommended (Class I, level of evidence B). In contrast, for patients at high risk of mortality, an initial triple combination therapy with a PDE5i, an ERA, and an intravenous or subcutaneous prostacyclin analog should be considered (Class IIa, level of evidence C). PAH patients should undergo regular follow-up to optimize their medical therapy. In fact, ESC guidelines recommend continuing the current therapy in patients who achieve and maintain a low-risk status. For those who remain at intermediate–low risk despite treatment with ERA and PDE5i, the addition of selexipag may be considered to reduce the risk of clinical worsening. In this subgroup, switching from PDE5i to riociguat may also be an option. In patients at intermediate–high or high risk despite oral therapy, escalation to intravenous epoprostenol or intravenous/subcutaneous treprostinil should be considered, along with referral to a specialized center for the evaluation of lung transplantation. If the initiation of intravenous or subcutaneous prostacyclin analogs is not feasible, adding selexipag or switching from PDE5i to riociguat may be considered as alternative strategies. In patients with PAH and concomitant cardiopulmonary comorbidities, initial monotherapy with a PDE5i or an ERA should be considered (Class IIa, level of evidence C). Although pharmacological therapy remains the first-line treatment, several invasive interventions have been developed in recent decades. The most recognized include the following:

- Pulmonary artery denervation, which uses radiofrequency ablation to disrupt stretch receptors at the pulmonary artery bifurcation, aiming to reduce vasoconstriction and vascular remodeling [26].
- Balloon atrial septostomy [27] and the Potts shunt [28], which aim to decompress the right heart and improve systemic perfusion.

While potentially beneficial, these invasive treatments should be considered only in specialized centers and reserved for symptomatic patients who remain refractory to optimal medical therapy. Finally, in selected patients for whom medical therapy proves inadequate, lung transplantation remains a last-resort option.

### 7.2. Group 2

In this group, PH is associated with left heart diseases. This includes patients with heart failure across its spectrum—heart failure with preserved ejection fraction (HFpEF), mildly reduced ejection fraction (HFmrEF), and reduced ejection fraction (HFrEF)—as well as those with valvular heart disease (e.g., mitral or aortic stenosis/regurgitation). For patients with PH due to left heart disease, treatment should target the underlying cardiac condition. This involves guideline-directed medical therapy for heart failure—such as beta-blockers, ACE inhibitors or ARNIs, and diuretics—as well as timely surgical or percutaneous intervention for valvular disease. In this context, PH is typically the result of the backward transmission of elevated left-sided pressures and pulmonary venous congestion. Therefore, pulmonary arterial hypertension (PAH)-specific vasodilators are not routinely indicated. Randomized trials evaluating the use of PAH-targeted therapies in PH due to left heart disease have generally shown no benefit—and, in some cases, harm. For example, studies on PDE5 inhibitors in HFpEF demonstrated no improvement in outcomes, and the MELODY-1 trial evaluating Macitentan in HFpEF-related PH was terminated early due to fluid retention. Management focuses on controlling volume overload, blood pressure, and myocardial ischemia. In cases where PH appears disproportionately severe relative to the degree of left-sided heart disease, advanced heart failure therapies may be considered. An exception may be patients with combined pre- and post-capillary PH who exhibit a significant pre-capillary component despite the optimal treatment of left heart disease. In such cases, referral to an expert center may be warranted for the discussion of the potential compassionate (off-label) use of PAH therapies. Ultimately, improving left heart function—whether through medical optimization, heart transplantation, or implantation of a ventricular assist device in advanced stages—remains the cornerstone for relieving PH in Group 2. Careful evaluation is particularly important in patients with mixed or overlapping etiologies. For instance, a patient with connective tissue disease may have both PAH and HFpEF. In these cases, addressing only the arterial component without managing elevated left-sided pressures would be inadequate.

### 7.3. Group 3

PH is frequently observed in patients with COPD, emphysema, interstitial lung disease (ILD), combined pulmonary fibrosis and emphysema (CPFE), and hypoventilation syndromes. It is more rarely seen in isolated obstructive sleep apnea, unless coexisting conditions such as COPD or daytime hypoventilation are present. Currently, there is insufficient evidence to support the routine use of PAH-specific therapies in patients with PH due to COPD or other lung diseases. The primary strategy in Group 3 PH is to treat the underlying respiratory disorder. This includes bronchodilators and inhaled corticosteroids for COPD, antifibrotic agents for idiopathic pulmonary fibrosis (IPF), continuous positive airway pressure (CPAP) for obstructive sleep apnea, and long-term oxygen therapy for chronic hypoxemia (aiming for SpO_2_ ≥ 90%). Based on the results of the INCREASE trial [29], the 2022 ESC guidelines recommend for the first time inhaled treprostinil as a potential treatment option for patients with PH associated with ILD (Class IIb, level of evidence B). The INCREASE trial demonstrated that inhaled treprostinil, a prostacyclin analog administered via nebulizer, improves exercise capacity in patients with PF-ILD and PH. Its selective vasodilatory action on well-ventilated lung regions enhances perfusion without causing significant systemic hypotension, making it a promising therapeutic strategy in ILD-PH. Beyond inhaled treprostinil, other PAH-targeted therapies have not shown clear benefit in COPD- or ILD-associated PH and are generally reserved for use in clinical trials. Patients with advanced lung disease and disproportionate PH should be evaluated for lung transplantation, where appropriate, as this may address both the pulmonary disease and associated PH. Supportive therapies such as pulmonary rehabilitation and structured exercise training are also essential, as they can significantly improve functional capacity and quality of life.

### 7.4. Group 4

This group includes PH due to CTEPH. Lifelong anticoagulation is recommended for all patients (Class I, level of evidence C). In patients with surgically accessible occlusive lesions of the pulmonary arteries, pulmonary endarterectomy (PEA) is the treatment of choice (Class I, level of evidence B). PEA is a highly specialized surgical procedure involving the removal of organized thromboembolic material from the pulmonary arteries, including segmental and subsegmental branches in experienced centers. In carefully selected candidates, PEA can significantly reduce pulmonary pressures, normalize hemodynamics, and is associated with a postoperative 3-year survival rate of approximately 90% [30]. Despite advances in surgical techniques, around 20–40% of CTEPH patients are considered inoperable due to distal disease or comorbidities. For these patients, two key alternatives are available: balloon pulmonary angioplasty (BPA) and medical therapy. BPA is recommended for patients who are technically inoperable or who have persistent/recurrent PH after PEA, with accessible distal lesions (Class I, level of evidence B). This percutaneous procedure uses balloon catheters to dilate the narrowed pulmonary vessels and restore perfusion. Refinements in technique—particularly by Japanese centers—have significantly improved the safety and outcomes of BPA, with procedure-related mortality now below 2% [31]. BPA has been shown to yield meaningful improvements in mean pulmonary artery pressure (often a drop of >10 mmHg), pulmonary vascular resistance, WHO functional class, and 6MWD through a series of staged interventions [32]. The only pharmacologic agent approved for CTEPH is riociguat, a soluble guanylate cyclase stimulator, indicated for symptomatic inoperable patients or those with persistent/recurrent PH after PEA (Class I, level of evidence B). Riociguat improves exercise capacity and hemodynamic parameters, and it is often used in combination with BPA or as a bridge to surgery. Other PAH drugs (e.g., ERAs, PDE5 inhibitors) are sometimes used off-label in expert centers, particularly when riociguat is not tolerated, or are used as add-on therapy, although robust evidence supporting their use in CTEPH is limited.

### 7.5. Group 5

Group 5 includes a heterogeneous collection of diseases associated with the complex, multifactorial mechanisms of PH. These conditions often involve overlapping vascular, parenchymal, and systemic contributions, making management particularly challenging. Treatment should be directed toward the underlying condition responsible for PH. For example, the following treatments are used:

- In sarcoidosis-associated PH, therapy includes immunosuppressive agents such as corticosteroids and general PH management measures.
- In sickle cell disease, optimal control of the hematologic disorder with hydroxyurea and transfusion therapy is key.

There are currently no approved PAH-specific therapies for Group 5. However, in selected cases—such as severe sarcoidosis-associated PH—specialized centers may consider the compassionate use of PAH treatments. Small studies have suggested possible hemodynamic benefits with agents like Bosentan or inhaled prostanoids in such contexts. Given the often multifactorial etiology of PH in Group 5, consultation with a PH expert center is strongly recommended to guide management, especially when considering off-label treatments. Ultimately, addressing modifiable contributors such as chronic anemia, metabolic disorders, or fibrosis may help reduce PH burden. In end-stage cases, transplantation (lung or heart–lung) may be an option, provided the underlying condition is amenable to transplant.

## 8. Pediatric Pulmonary Hypertension

PH can affect individuals of all ages, including the pediatric population. In children, early diagnosis is crucial to prevent irreversible damage to the developing fetal, neonatal, or pediatric pulmonary circulation. The most common form of PH in children is PAH. This includes transient conditions such as persistent pulmonary hypertension of the newborn (PPHN) and PAH secondary to repairable congenital heart defects. Other forms include idiopathic PAH (IPAH), heritable PAH (HPAH), and irreversible PAH associated with congenital heart disease (CHD-PAH). A subset of neonates and infants may develop non-transient PH due to developmental lung diseases such as bronchopulmonary dysplasia (BPD), congenital diaphragmatic hernia (CDH), and congenital pulmonary vascular abnormalities. The 6th and 7th World Symposium on Pulmonary Hypertension (WSPH) pediatric task forces emphasized that the general definition of PH (mPAP > 20 mmHg in children over 3 months of age) is applied for consistency [33] with adult definitions. However, neonatal PH within the first weeks of life—such as PPHN—is considered a distinct clinical entity, often with a transient course. Certain conditions, such as PPHN or lung developmental disorders, are included exclusively in the pediatric PH classification. Diagnosis in children relies primarily on echocardiography and clinical evaluation, as symptoms like failure to thrive or fatigue may be subtle or masked by comorbidities. RHC is mandatory for confirming the diagnosis of PH and for conducting acute vasoreactivity testing, especially in children [34] with IPAH or HPAH. A positive response may identify candidates for CCB therapy. However, in children with CHD-associated PAH, these criteria do not define disease reversibility. Genetic testing is strongly recommended in pediatric PAH, as the diagnostic yield is high and results have important implications for family counseling. For example, a BMPR2 mutation warrants surveillance in relatives, while a TBX4 mutation may suggest concurrent skeletal or lung developmental anomalies. Risk stratification in children has evolved, recognizing that children are not simply “small adults.” Pediatric-specific factors—such as growth parameters, developmental milestones, and age-appropriate functional assessments—must be considered. Approximately 25 variables have been identified for pediatric risk assessment, many adapted from adult models but adjusted for pediatric norms. These include the following:

- WHO functional class (adapted for age);
- Growth failure;
- Episodes of syncope;
- BNP/NT-proBNP levels;
- Echocardiographic measures of right ventricular function;
- Hemodynamic indices;
- Genetic markers.

Although pediatric risk models are less validated than those for adults, there is a consensus that children with preserved growth, normal or low BNP levels, good functional class, and maintained right ventricular function have a better prognosis. Risk re-evaluation every 3–6 months is recommended to guide therapeutic decisions. The treatment algorithm for pediatric PAH is based on adult strategies but adapted for age, comorbidities, and developmental stage. The 2024 pediatric PH guidelines (7th WSPH) introduced a dual-arm treatment algorithm:

- Children without cardiopulmonary comorbidities (e.g., idiopathic/heritable PAH or PAH associated with systemic diseases);
- Children with cardiopulmonary developmental disorders (e.g., congenital heart disease, lung developmental abnormalities).

This distinction reflects the need for individualized treatment approaches. Children without significant comorbidities can often be treated more aggressively with PAH-targeted therapies, while those with complex cardiac or pulmonary conditions may require combined medical and surgical strategies. For acute vasoreactivity responders, initial treatment with CCBs is appropriate, though only a minority of children qualify. For non-responders, combination therapy with an ERA and PDE5 inhibitor is recommended, with the addition of a prostacyclin analog if necessary to achieve and maintain a low-risk profile. While many PAH drugs are used off-label in pediatrics, several have pediatric formulations or established dosing protocols. These include the following:

- Bosentan (ERA);
- Sildenafil (PDE5 inhibitor);
- Inhaled iloprost;
- Subcutaneous treprostinil.

For children with underlying heart or lung disease, treatment should also address the primary condition. This may include the following:

- Corrective surgery for congenital shunts;
- Pulmonary therapies for lung disorders.

In advanced or refractory cases, pediatric-specific interventions may be necessary. These include the following:

- Atrial septostomy;
- Potts shunt (a surgical connection between the left pulmonary artery and descending aorta), which can decompress the right heart and improve survival in selected children with suprasystemic PAH.

Lung transplantation, or heart–lung transplantation in cases with complex cardiac anomalies, remains a last-resort option. Timely referral to pediatric transplant centers is critical when combination therapy fails to halt disease progression.

## 9. Emerging Therapies in PAH

Advances in the understanding of the pathophysiological mechanisms underlying PAH have led to the development of several novel therapeutic strategies, particularly for patients in WHO Group 1. Most of the available data on these emerging treatments derive from Phase II or III randomized controlled trials.

- Sotatercept is a first-in-class biologic therapy as an activin receptor IIA-Fc fusion protein that modulates signaling in the BMP/TGF-β superfamily. By binding activins and growth differentiation factors, sotatercept aims to rebalance the dysregulated pro-proliferative TGF-β pathway seen in PAH and restore BMPR2 signaling. In the pivotal STELLAR trial, sotatercept—used in addition to background PAH therapy—showed significant clinical benefit, with a +34 m increase in 6 min walk distance at 24 weeks compared to placebo, along with improvements in 8 out of 9 secondary endpoints (including PVR, NT-proBNP levels, and WHO functional class). This marks a major advance, as sotatercept acts at a molecular level, targeting vascular remodeling rather than simply vasodilation. The therapy was generally well tolerated, with mostly mild adverse effects (e.g., epistaxis, telangiectasias, elevated hemoglobin, thrombocytopenia) related to its mechanism of action. Sotatercept is currently under further investigation in ongoing trials (e.g., HYPERION and ZENITH) and may soon represent a disease-modifying option in PAH, potentially transforming the treatment paradigm in combination with current vasodilators.
- Ralinepag is a next-generation oral prostacyclin receptor agonist (non-prostanoid), developed to provide potent, sustained activation of the prostacyclin pathway. With a high affinity for the IP receptor and a long plasma half-life, it is designed for once-daily dosing to improve treatment adherence and convenience. Phase II studies indicate that ralinepag improves hemodynamic parameters, and it is currently being evaluated in the ADVANCE program (Phase III), including the ADVANCE-Outcomes trial, which is assessing its ability to delay disease progression and enhance exercise capacity when added to PDE5i/ERA-based therapy. If successful, ralinepag could offer a practical oral alternative to parenteral prostacyclin analogs and support the earlier adoption of triple combination therapy. Although results are pending (as of 2024), optimism is high due to the already validated IP receptor pathway (e.g., selexipag).
- Inhaled imatinib (dry powder formulation), a tyrosine kinase inhibitor initially used in chronic myeloid leukemia, demonstrated a significant reduction in PVR in a Phase III PAH trial over a decade ago. However, its oral administration was limited by systemic side effects (e.g., peripheral edema, subdural hematomas). This led to the development of inhaled imatinib (e.g., AV-101), a dry powder formulation designed to deliver the drug directly to the pulmonary vasculature, thereby reducing systemic exposure. The IMPAHCT trial [35], an ongoing Phase IIb/III study, is evaluating its efficacy and safety. Inhaled imatinib aims to reverse vascular remodeling by inhibiting PDGF and c-Kit signaling in the lungs. Preliminary data suggest a better safety profile than the oral form, although optimal dosing is still under investigation. If successful, it could represent a repurposed cancer drug with targeted disease-modifying potential for advanced PAH.
- New Treprostinil formulations: Treprostinil, a prostacyclin analog, has long been a cornerstone of PAH therapy, typically administered via intravenous, subcutaneous, or inhaled routes. Recent innovations aim to improve patient convenience and safety, expanding access to prostacyclin-based treatment. Tyvaso DPI (dry powder inhaler): Approved by the Food and Drug Administration in 2022 for PAH (Group 1) and PH-ILD (Group 3) [36]. Tyvaso DPI delivers Treprostinil via a breath-actuated handheld device eliminating the need for nebulizers or external power sources. Clinical studies showed comparable efficacy to nebulized Treprostinil, with high patient satisfaction and no new safety concerns. It allows more convenient, outpatient-friendly administration, including in patients with comorbid lung disease. Implantable Treprostinil pump: Already in use in Europe, this device provides continuous IV Treprostinil infusion while reducing infection risk associated with external catheter lines, improving long-term safety. Treprostinil Palmitil: An inhalable prodrug formulation of Treprostinil designed for once-daily administration, currently under investigation. It may offer ultra-long-acting prostacyclin activity, further simplify dosing and improving adherence. These innovations aim to retain the robust efficacy of Treprostinil while enhancing tolerability and accessibility across different stages of disease.
- Several other investigational agents show potential as adjunctive or disease-modifying treatments: Seralutinib (inhaled): A PDGF receptor kinase inhibitor, currently under evaluation in the TORREY trial for PAH, targeting vascular remodeling pathways. Rodatristat ethyl: An oral tryptophan hydroxylase inhibitor that reduces peripheral serotonin levels—a known mediator of pulmonary vascular proliferation in PAH. Immunomodulatory agents: Including anastrozole, which reduces estrogen levels implicated in PAH pathogenesis, and tocilizumab, an IL-6 receptor antagonist under investigation for CTD-associated PAH.

These emerging therapies represent a shift in the PAH treatment landscape—from primarily vasodilator-based approaches to more pathophysiologically targeted interventions. With promising data from ongoing trials, these agents hold the potential to modify disease progression, improve long-term outcomes, and personalize treatment strategies in PAH.

## 10. Prognosis and Follow-Up Strategies

The prognosis of PH has improved significantly in the modern treatment era, though it remains highly dependent on the underlying PH group and the risk profile achieved during therapy. For example, idiopathic pulmonary arterial hypertension (IPAH) now has an estimated 5-year survival rate of 60–70%, compared to approximately 40% two decades ago [37]. Patients diagnosed early and who achieve a low-risk status through therapy have the most favorable outcomes, sometimes approaching near-normal life expectancy. Conversely, patients who remain at high risk—for instance, those in WHO functional Class IV with severe right ventricular (RV) dysfunction—face poor survival rates, with a 1-year mortality exceeding 20%, as documented in registry data. Prognosis in other forms of PH is closely tied to the underlying disease. For example, patients with CTEPH who undergo successful PEA may be effectively cured and enjoy a normal lifespan. In contrast, those with Group 3 PH due to advanced interstitial lung disease often have a high mortality risk unless lung transplantation is performed. In pediatric PAH, survival has also improved in recent years. Historically, outcomes were worse than in adults, due in part to delayed diagnosis and more aggressive disease forms. Fortunately, modern treatments now enable many children to survive into adolescence and adulthood, with registries reporting 1-year survival rates exceeding 90% in treated pediatric populations. Risk stratification tools play a central role in guiding follow-up and treatment decisions. The overarching goal is to achieve and maintain a low-risk profile. Follow-up visits should include the regular assessment of a combination of parameters:

- WHO functional class;
- Exercise capacity (6MWD or Cardiopulmonary Exercise Testing, when feasible);
- Biomarkers (particularly NT-proBNP);
- Imaging (echocardiographic assessment of RV size and function; some centers utilize serial cardiac MRI);
- Hemodynamic measurements (via periodic RHC, particularly when considering transplant referral).

Validated tools like the REVEAL 2.0 risk score (based on U.S. registry data) and the ESC/ERS 3- or 4-strata risk assessment models can quantitatively assess mortality risk at each visit, classifying patients into low, intermediate (with possible subdivisions), or high risk of 1-year mortality [37]. A key concept is the “treat-to-target” approach: if a patient is not in the low-risk category, therapy should be escalated—when possible—until low-risk criteria are met, or until maximal medical therapy has been reached. At that point, referral for lung transplantation evaluation should be considered in refractory cases. Follow-up intervals are generally every 3 to 6 months for stable patients, and more frequently during treatment initiation or adjustment. During these visits, in addition to risk assessment, clinicians monitor the following:

- Adverse effects of therapy;
- Routine laboratory values (e.g., liver function tests in patients on endothelin receptor antagonists);
- Comorbid conditions such as sleep apnea, anemia, or iron deficiency;
- Patient adherence and education, including recognition of worsening symptoms.

A growing area of interest is the use of telemedicine and remote monitoring to detect early signs of clinical deterioration. Technologies under investigation include the following:

- Wearable devices and smartphone applications to track heart rate, oxygen saturation, and daily physical activity.
- Integration of smartwatch data (heart rate, step count, heart rate variability) directly into electronic health records for physician review [38].
- Trials exploring whether declines in activity levels or rising resting heart rates can predict decompensation before clinical symptoms appear.

While not yet the standard of care, these tools may soon complement traditional follow-up strategies by providing continuous, real-world physiological data. In selected patients, implantable hemodynamic monitors (such as CardioMEMS, already approved for heart failure) are being explored for PH management, particularly in those with overlapping left heart disease [39].

Comprehensive follow-up also includes the following:

- Supervised exercise training, which has been shown to improve functional capacity and quality of life in PAH.
- Psychological support, as many patients suffer from anxiety or depression due to chronic illness; referrals to mental health services and support groups are often beneficial.
- Palliative care, which can be introduced at any stage of advanced PH to address symptom burden and improve quality of life—not limited to end-stage disease.

A central goal of follow-up is to optimize therapy to slow disease progression and anticipate complications—particularly the need for lung transplantation—before the disease becomes end-stage. For instance, signs of right heart failure or rising NT-proBNP levels should trigger early transplant referral in eligible patients. Multidisciplinary management is essential, involving cardiologists, pulmonologists, rheumatologists (for connective tissue disease-associated PH), and specialized nurses or physician assistants with expertise in PH. Thanks to aggressive treatment and structured follow-up strategies, many patients with PH now experience improved functional capacity, better quality of life, and longer survival. However, vigilance remains crucial, as the disease can progress insidiously. Risk-based follow-up—enhanced by novel technologies and a multidisciplinary approach—is key to achieving the best possible outcomes in this complex and heterogeneous disease.

## 11. Role of Expert Centers in Pulmonary Hypertension Management

Across all aspects of PH care—diagnosis, treatment, and follow-up—the involvement of specialized PH centers (also known as comprehensive care centers) is strongly endorsed by international guidelines. PH is a complex, multisystemic disease requiring coordinated, multidisciplinary management. Outcomes are consistently better when patients are treated in centers with dedicated expertise and infrastructure. These expert centers typically provide integrated care from a team of specialists, including cardiologists and pulmonologists experienced in PH, rheumatologists (particularly for connective tissue disease-associated PH), advanced practice providers, pharmacists, and social workers—all trained in the nuances of PH management. They also have access to advanced diagnostic tools, such as the following:

- Specialized ETT with PH-specific protocols.
- RHC (including provocative maneuvers like exercise or fluid challenge when appropriate).
- Cardiopulmonary Exercise Testing (CPET).
- Imaging modalities such as ventilation/perfusion SPECT and cardiac MRI.

Perhaps most critically, these centers are able to initiate and manage therapies that are not routinely available in community settings, including the following:

- Intravenous prostacyclin therapy.
- Titration of complex drug regimens.
- Access to clinical trials of emerging therapies.
- Multidisciplinary evaluation for lung or heart–lung transplantation.

International guidelines now emphasize the need for “fast-track” referral pathways for patients with suspected PH [40]. Any patient with an intermediate or high echocardiographic probability of PH, or with confirmed PH of unclear etiology, should be referred without delay to an expert center for comprehensive workup, including hemodynamic confirmation via right heart catheterization. Early referral is especially crucial for patients with PAH or CTEPH, where timely intervention can significantly improve outcomes—for example, with the early initiation of combination therapy in PAH or operability assessment and potential surgery in CTEPH. Studies have shown that delayed referral is associated with poorer prognosis, as untreated or undertreated patients may progress to irreversible right ventricular failure. In contrast, data from national and international PH registries consistently demonstrate superior survival among patients managed at PH centers. For example, one international registry reported 3-year survival rates as high as 89% among PAH patients under expert center care, reflecting the benefits of aggressive, tailored treatment and close monitoring. From the patient’s perspective, PH centers offer comprehensive support, including the following:

- Patient education on lifestyle modifications and symptom recognition;
- Vaccination programs (e.g., for influenza and pneumococcal infections);
- Infection prophylaxis;
- 24/7 on-call services, which are essential for patients on continuous infusions or with implantable devices.

Regular multidisciplinary meetings allow for detailed discussion of complex cases—such as patients with overlapping PH etiologies (e.g., combined Group 2 and 3 PH) or multiple comorbidities—ensuring that individualized care plans are developed and adapted over time. Expert centers are also typically active in clinical research, keeping pace with novel therapeutic strategies and offering patients early access to promising treatments when standard options are exhausted. The center of excellence model concentrates clinical expertise and supports national and regional referral networks, linking local providers to PH specialists. In recognition of their critical role, some countries have established formal accreditation systems for PH centers, based on defined criteria such as clinical experience, multidisciplinary staffing, and infrastructure. Finally, the global PH community benefits from the work of national and international scientific societies [41], which support working groups, consensus statements, and local position papers, helping to harmonize care and guide best practices worldwide. In 2025, the management of PH is unequivocally a specialized field. Optimal outcomes—in terms of both survival and quality of life—are closely tied to early referral to and ongoing follow-up at PH expert centers. These centers not only deliver advanced medical care but also provide patients with the confidence that their complex condition is being managed by a dedicated, experienced, and multidisciplinary team. Their role is central to the continued progress in PH care, and they serve as key drivers of innovation, education, and quality improvement in the field.

## 12. Conclusions

PH remains a complex and progressive condition that sits at the intersection of cardiology and pulmonology. However, recent years have witnessed significant progress in both our understanding and management of the disease. Epidemiological data underscore the importance of early detection, particularly in at-risk populations such as patients with connective tissue disease or a history of pulmonary embolism. The updated hemodynamic definition of PH now allows for earlier diagnosis, potentially before irreversible vascular damage occurs. Advancements in risk stratification and a renewed emphasis on frequent reassessment have led to a more proactive, dynamic therapeutic approach—aimed at maintaining or achieving a low-risk profile and preventing clinical deterioration. Innovative diagnostic tools—such as ventilation/perfusion SPECT and perfusion MRI—have improved our ability to detect and characterize PH, particularly in CTEPH, with greater precision. On the scientific front, breakthroughs in understanding the molecular and inflammatory pathways of PH (e.g., BMPR2 mutations, TGF-β signaling, and vascular remodeling) are being translated into targeted therapies. Agents like sotatercept, which aims to modify disease progression by reversing vascular remodeling, mark a shift from symptomatic to disease-modifying treatment strategies. In clinical practice, a structured, phenotype-driven approach based on PH classification (Groups 1–5) enables individualized therapy:

- Aggressive combination treatment in Group 1 PAH.
- Surgical or interventional correction for Groups 2 and 4.
- Supportive, symptom-directed care in Groups 3 and 5, with selective use of novel therapeutic options.

Emerging therapies such as sotatercept, ralinepag, and inhaled imatinib, as well as user-friendly formulations like Tyvaso DPI, reflect a rapidly evolving field that is focused not only on improving survival but also on enhancing patient quality of life. Notably, the progress in CTEPH management—with increased success in both pulmonary endarterectomy and balloon pulmonary angioplasty—is transforming outcomes in what was once a highly morbid condition. Perhaps the most critical theme in PH care today is the central role of expert centers and multidisciplinary collaboration. From diagnosis to advanced therapies and transplant evaluation, specialized teams ensure that patients receive the most up-to-date, evidence-based, and personalized care. As we continue to incorporate new scientific discoveries into clinical practice, the commitment to expert, risk-guided, and patient-centered management will remain the cornerstone of improving outcomes in this challenging but increasingly treatable disease.

## Abbreviations

The following abbreviations are used in this manuscript:

6MWD	Six-minute walking distance
BPA	Balloon pulmonary angioplasty
BMPR2	Bone morphogenetic protein receptor type 2
BNP	Brain natriuretic peptide
CCBs	Calcium channel blockers
CPET	Cardiopulmonary Exercise Testing
ppm	Cases per million
CLD	Chronic lung disease
COPD	Chronic obstructive pulmonary disease
CTEPH	Chronic thromboembolic pulmonary hypertension
CPFE	Combined pulmonary fibrosis and emphysema
CHD	Congenital heart disease
CPAP	Continuous positive airway pressure
DECT	Dual-energy computed tomography
ERAs	Endothelin receptor antagonists
ESC	European Society of Cardiology
HF	Heart failure
HFmrEF	Heart failure with mildly reduced ejection fraction
HFpEF	Heart failure with preserved ejection fraction
HFrEF	Heart failure with reduced ejection fraction
HPAH	Heritable PAH
IPAH	Idiopathic PAH
IPF	Idiopathic pulmonary fibrosis
IL-6	Interleukin-6
ILD	Interstitial lung disease
LHD	Left heart disease
mPAP	Mean pulmonary arterial pressure
NO	Nitric oxide
PDE5i	Phosphodiesterase type 5 inhibitor
PDGF	Platelet-derived growth factor
PAs	Prostacyclin analogs
PRAs	Prostacyclin receptor agonists
PAH	Pulmonary arterial hypertension
PAWP	Pulmonary artery wedge pressure
PEA	Pulmonary endarterectomy
PH	Pulmonary hypertension
PPHN	Pulmonary hypertension of the newborn
PVR	Pulmonary vascular resistance
PVOD	Pulmonary veno-occlusive disease
RHC	Right heart catheterization
sGCS	Soluble guanylate cyclase stimulator
TGF-β	Transforming growth factor-beta
V/Q	Ventilation/perfusion
WU	Wood Units
WHO	World Health Organization
WSPH	World Symposium on Pulmonary Hypertension
